# SOCIAL DETERMINANTS OF SMOKING CESSATION IN MIDDLE-AGED AND OLDER ADULTS WITH SERIOUS MENTAL ILLNESSES

**DOI:** 10.1093/geroni/igad104.0674

**Published:** 2023-12-21

**Authors:** Heather Leutwyler, Erin Hubbard, Theodore Bussell, Negin Zahedikia, Dennys Balestra, Margaret Wallhagen, Chizimuzo Okoli

**Affiliations:** University of California, San Francisco, San Francisco, California, United States; UCSF, San Francisco, California, United States; University of San Francisco School of Nursing, San Francisco, California, United States; University of California San Francisco, San Francisco, California, United States; University of California, San Francisco, San Francisco, California, United States; University of California, San Francisco, San Francisco, California, United States; University of Kentucky, Lexington, Kentucky, United States

## Abstract

People living with serious mental illnesses (SMI) continue to face a disproportionate burden of tobacco-related prevalence, morbidity, and mortality as compared to those without SMI. The risk of mortality related to cigarette smoking among those with SMI is six-times that for persons without SMI. Tobacco use undermines social determinants of health (SDoH) in general and the social determinants of mental health more specifically. Not only does tobacco use exacerbate mental health symptoms, it further impedes mental health recovery by reducing expendable income, sustaining poverty, hindering food security, and limiting job opportunities. Conversely, stopping tobacco use is associated with improved mental health and, possibly, substance use recovery outcomes. The purpose of this paper is to analyze the social determinants of tobacco smoking cessation as described by adults with SMI. We conducted semi-structured qualitative interviews with 17 middle aged and older adults (mean=52, sd=6.9) with SMI upon completion of a 12-week smoking cessation program. Grounded Theory methodology guided data collection and analysis. Participants described what contributed to their success with smoking cessation or reduction and pointed out how SDoH played a role in the process. Five broad categories of SDoH described were: 1. Support and sense of belonging; 2. Structure and meaningful activities; 3. Employment; 4. Role models; and 5. Access to green space. Our findings illustrate the importance of SDoH in health interventions and are a reminder to consider these SDoH as interventions are tailored to meet the unique needs of people living with SMI.

